# Supernumerary proteins of the human mitochondrial ribosomal small subunit are integral for assembly and translation

**DOI:** 10.1016/j.isci.2024.110185

**Published:** 2024-06-04

**Authors:** Taru Hilander, Ryan Awadhpersad, Geoffray Monteuuis, Krystyna L. Broda, Max Pohjanpelto, Elizabeth Pyman, Sachin Kumar Singh, Tuula A. Nyman, Isabelle Crevel, Robert W. Taylor, Ann Saada, Diego Balboa, Brendan J. Battersby, Christopher B. Jackson, Christopher J. Carroll

**Affiliations:** 1 Genetics Section, Cardiovascular and Genomics Research Institute, St George’s, University of London, London, UK; 2 Department of Biochemistry and Developmental Biology, Faculty of Medicine, University of Helsinki, Helsinki, Finland; 3 Stem Cells and Metabolism Research Program, Faculty of Medicine, University of Helsinki, 00290 Helsinki, Finland; 4 Department of Immunology, Institute of Clinical Medicine, University of Oslo and Oslo University Hospital, Oslo, Norway; 5 Core Facilities, St George’s, University of London, London, UK; 6 Mitochondrial Research Group, Translational and Clinical Research Institute, Faculty of Medical Sciences, Newcastle University, Newcastle upon Tyne NE2 4HH, UK; 7 NHS Highly Specialised Service for Rare Mitochondrial Disorders, Newcastle Upon Tyne Hospitals NHS Foundation Trust, Newcastle upon Tyne NE1 4LP, UK; 8 Department of Genetics, Hadassah Medical Center & Faculty of Medicine, Hebrew University of Jerusalem, Jerusalem 9112001 Israel; 9 Institute of Biotechnology, HiLIFE, University of Helsinki, Helsinki, Finland

**Keywords:** biological sciences, biochemistry, molecular biology, cell biology

## Abstract

Mitochondrial ribosomes (mitoribosomes) have undergone substantial evolutionary structural remodeling accompanied by loss of ribosomal RNA, while acquiring unique protein subunits located on the periphery. We generated CRISPR-mediated knockouts of all 14 unique (mitochondria-specific/supernumerary) human mitoribosomal proteins (snMRPs) in the small subunit to study the effect on mitoribosome assembly and protein synthesis, each leading to a unique mitoribosome assembly defect with variable impact on mitochondrial protein synthesis. Surprisingly, the stability of mS37 was reduced in all our snMRP knockouts of the small and large ribosomal subunits and patient-derived lines with mitoribosome assembly defects. A redox-regulated CX_9_C motif in mS37 was essential for protein stability, suggesting a potential mechanism to regulate mitochondrial protein synthesis. Together, our findings support a modular assembly of the human mitochondrial small ribosomal subunit mediated by essential supernumerary subunits and identify a redox regulatory role involving mS37 in mitochondrial protein synthesis in health and disease.

## Highlights

• Generation of 14 supernumerary mitochondrial ribosomal proteins knockouts (snMRP^KO^s)
• snMRPs of the small mitoribosomal subunit (SSU) are essential structural proteins
• snMRP^KO^s allow to determine mitoribosomal assembly intermediates
• mS37 is a regulator of mitochondrial protein synthesis

## Graphical abstract

**Figure:**
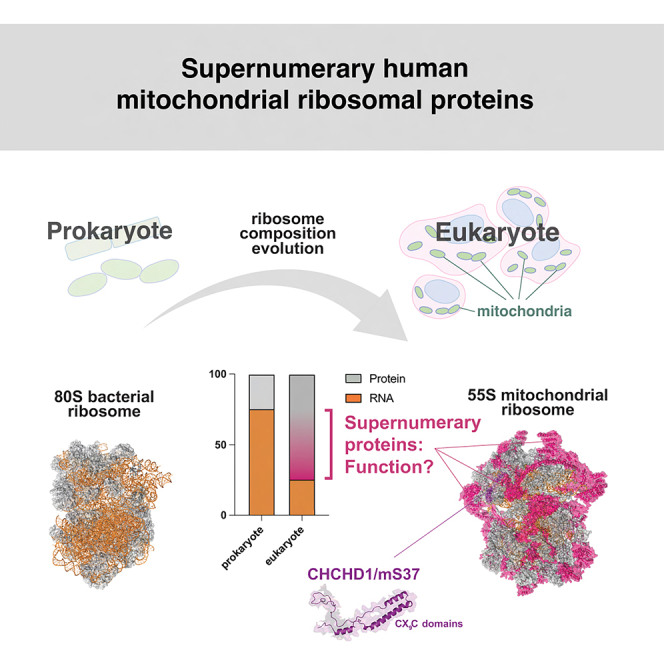

## Introduction

Mitochondria are cellular organelles of prokaryotic origin where ATP is synthesized by oxidative phosphorylation (OXPHOS). While most mitochondrial genes have been transferred to the nucleus during evolution, human mitochondria continue to harbor their own compacted circular genome. Human mitochondrial DNA (mtDNA) encodes 13 highly hydrophobic structural subunits of the OXPHOS enzyme complexes, while the remaining genes encode 22 tRNAs and 2 rRNAs required for mitochondrial protein synthesis.^1^^,^^2^^,^^3^^,^^4^^,^^5^^,^^6^^,^^7^^,^^8^

The mature human mitoribosome (55S) is composed of a small (28S, mtSSU) and a large (39S, mtLSU) subunit.^5^^,^^6^^,^^9^ The human mtSSU is composed of 30 proteins and a 12S rRNA, while the mtLSU consists of 52 proteins, a 16S rRNA, and a copy of mitochondrial valine tRNA.^10^ Across the eukaryotic taxa, mitoribosomes differ in size and the protein-to-RNA ratio, including mammals (55S), budding yeast (74S), and plants (78S) (Figure S1).^2^^,^^7^^,^^8^^,^^11^^,^^12^ The increase in mitoribosomal protein abundance originates from increased extensions in shared homologous proteins and the addition of mitochondrial-specific proteins, also known as supernumerary mitochondrial ribosomal proteins (snMRPs).^13^ The recent cryogenic-electron microscopy (cryo-EM) structures of mitoribosomes from human, porcine, and yeast reveal that the supernumerary proteins are extensions occupying completely new positions, rather than a replacement of lost rRNA segments.^5^^,^^7^^,^^8^ It is currently unclear whether these snMRPs primarily compensate for the reduced rRNA abundance or participate in additional functions important for mitochondrial protein synthesis. With the growing number of reports of human inherited diseases with mitochondrial protein synthesis defects and tissue-specific symptoms, including several mitoribosomal subunit proteins (Table S1), it is important to understand the role of these snMRPs.

The aim of our study was to determine the role of snMRPs for assembly of the human mtSSU and function in mitochondrial protein synthesis. We used genome editing to generate knockout cell lines for the snMRPs of the human mtSSU. Collectively, we find distinct phenotypes for each of the snMRPs on mitoribosome assembly and protein synthesis, in addition to identifying a role for mS37 in regulation of mitochondrial protein synthesis.

## Results

### CRISPR-mediated knockout of snMRPs of the mtSSU

**Figure 1:**
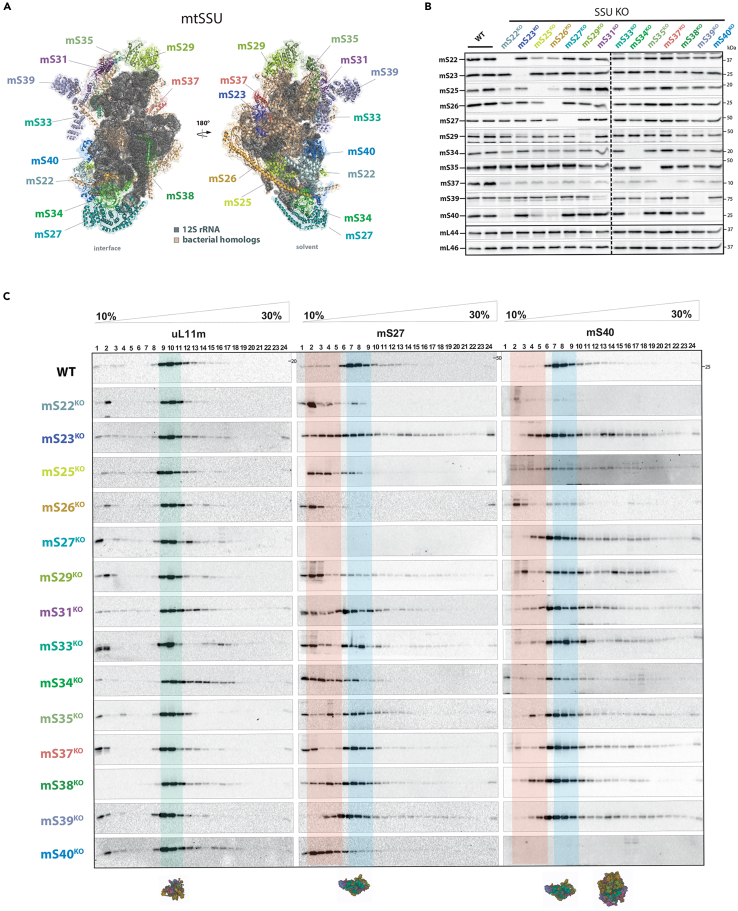
CRISPR-Cas9-mediated knockout of supernumerary proteins (snMRPs) of the human small mitoribosomal subunit (mtSSU) and effect on mitoribosome assembly (A) snMRPs of the mtSSU (28S). Denomination of cryo-EM resolved mammalian mtSSU structure (PDB: 5AJ3) highlighting snMRPs of the mtSSU. Bacterial homologs in orange, 12S RNA in dark gray. (B) Immunoblot confirmation of knockout in HEK293 cells. The dotted lines indicate the border of separate immunoblots. (C) Isokinetic sucrose gradients of all 14 snMRP knockouts reveal mtSSU assembly defects. Fully assembled mtLSU fractions (green); fully assembled mtSSU fractions 6–8 (blue); intermediates and loss of assembly (orange).

We used CRISPR-Cas9 to generate knockout cell lines for each of the human snMRPs in HEK293. The nomenclature for mitoribosomal subunit proteins that has been adopted in recent studies was applied here (Figure 1A).^5^^,^^8^^,^^14^ Since snMRPs are distributed on the periphery of the mtSSU and mtLSU—positions not previously occupied by the structural rRNA present in the bacterial ribosome—these proteins could have acquired additional mitochondrial-specific interactions for protein synthesis (Figure S2). We designed two guide RNAs (gRNAs) to all supernumerary proteins in the mtSSU: mS22, mS23, mS25, mS26, mS27, mS29, mS31, mS33, mS34, mS35, mS37, mS38, mS39, and mS40. The strategy was to excise a region encompassing the start codon, while ensuring no alternative initiation codons were present (key resources table). We successfully obtained clonal knockout cell lines for all 14 snMRPs of the mtSSU (Figure S3). All snMRP^KO^ cells were supplemented with uridine and pyruvate to bypass any potential growth defect caused by respiratory chain deficiency. Immunoblotting confirmed complete knockout in all snMRP^KO^ cells and revealed that each knockout had variable effects on the abundance of other mtSSU subunits (Figure 1B). Commercial antibodies were unavailable for mS31, mS33, and mS38, but the knockout allele was confirmed by PCR (Figure S3). In contrast to other mtSSU proteins, mS37 was uniformly decreased in all snMRP^KO^ cell lines, suggesting that, among mtSSU proteins, mS37 protein is uniquely regulated.

Next, we assessed the status of assembled mitoribosomal subunits in snMRP^KO^ cell lines. Isokinetic sucrose gradients revealed a unique pattern of mtSSU assembly defects for each of the snMRP^KO^ cell lines (Figure 1C), using antibodies against early (mS27) and intermediate (mS40) assembling MRPs (Figure S4). The stabilities of the small mitoribosomal proteins mS27 and mS40 have been identified as markers for the stepwise assembly from the early to intermediate stage, respectively.^15^ In our snMRP^KO^ cell lines, we observed a shift toward an intermediate assembly stage that was most prominent in the mS22^KO^, mS26^KO^, mS34^KO^, and mS40^KO^ cell lines using antibodies to report early- and late-assembled proteins mS27 and mS40. Assembly of the mtLSU was predominantly unaffected in our snMRP knockout cell lines when probed for uL11m (Figure 1C).

### Quantitative label-free proteomics reveals the impact on abundance of mtSSU proteins in snMRP knockouts

**Figure 2:**
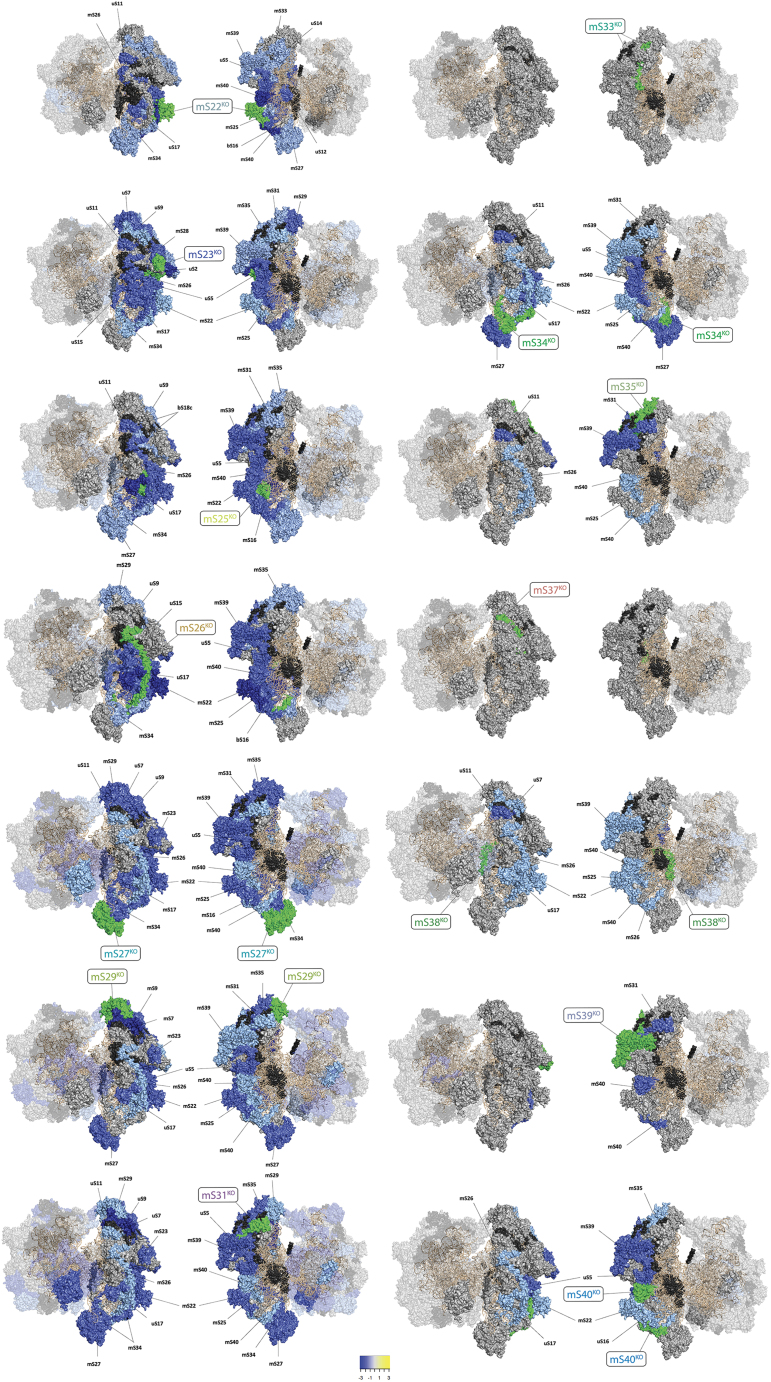
Proteomic expression mapping of the snMRP^KOs^ onto the 55S mitoribosome structure Ribosome structures based on (PDB entry: 5AJ4) and visualized using Pymol 2.7.1. Proteomic cutoff threshold for visualization was 1.5-fold change from wild type. For each snMRP^KO^ two images are shown rotated 180° including both mtSSU and mtLSU. The snMRP^KO^ cell line is indicated in green and labeled in a rectangular box. Proteins with differential expression are labeled. A gradient of blue/yellow colors indicates level of decrease/increase in expression (scale shown). Ribosomal RNA is orange. Proteins not changed in expression in gray. Proteins not reliably measured in black.

Next, we performed whole-cell quantitative label-free liquid chromatography-tandem mass spectrometry (LC-MS/MS) proteomic analysis for our snMRP^KO^ cell lines to assess the abundance of mtSSU proteins. We visualized the fold changes for all 14 snMRP^KO^ cell lines by mapping of the steady-state level with a 1.5-fold-expression change cutoff to the structure of the assembled 55S mitoribosome (Figure 2). Each snMRP^KO^ had a unique impact on the stability of other mtSSU proteins, which largely reflected the outcome for the assembled mtSSU observed in isokinetic sucrose gradients (Figure 1C). In whole-cell lysates of snMRPKO cells lines, where extensive mtSSU assembly defects were observed, also proteins of the mtLSU were significantly impacted, suggesting that SSU defects affect LSU assembly or steady-state protein levels (Figure 2). For the mS37^KO^ cell line there was no significant change in abundance of mtSSU proteins, corroborating the observation from the sucrose gradient experiments. This suggests that the abundance of mS37 does not affect mtSSU assembly. Importantly, the timing of protein incorporation during mtSSU assembly does not appear to be a reliable predictor for the steady-state abundance of the mtSSU.^15^ For example, mS25 and mS26 are known to be incorporated in the late stages of mtSSU assembly (Figure S4), and yet our knockout cell lines for these subunits had the most deleterious impact on the mtSSU abundance. Interestingly, even proteins that do not bind the 12S rRNA, such as mS22, can generate a profound assembly defect for the mtSSU. Together, these data show that knockout of snMRPs causes mtSSU assembly defects which are unique in each individual snMRP^KO^ cell line.

### Hierarchical clustering of mtSSU protein expression reveals assembly modules

**Figure 3:**
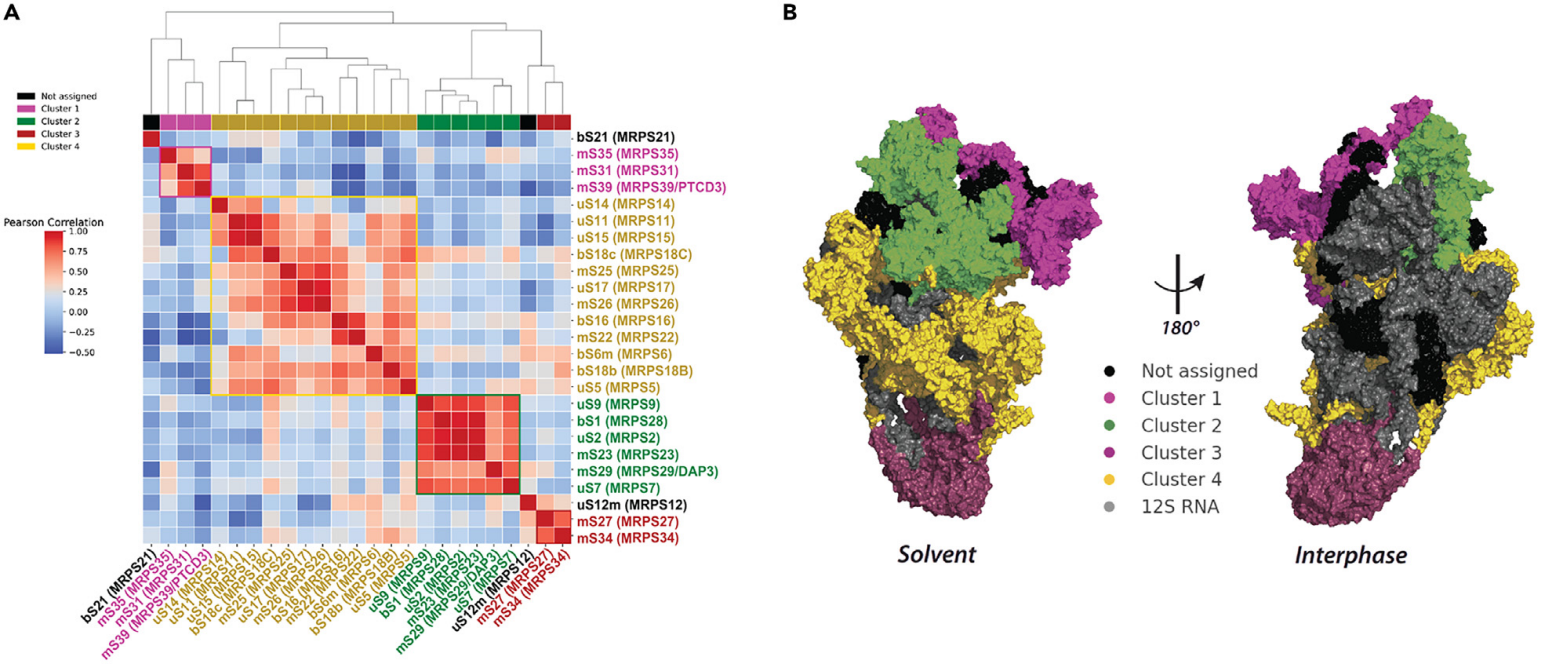
Hierarchical clustering of mtSSU protein expression reveals the composition of assembly modules of the mtSSU (A) Hierarchical clustering of Pearson correlation coefficients determined from the log-fold expression of all detected (25/29) MRPs. The heatmap colors indicate the Pearson correlation coefficient among the MRPs, with darker colors representing stronger correlations. The color bars show the identified assembly nodes. (B) Mapping of detected MRPs clusters on the mtSSU structure (PDB: 5AJ3). Not assigned proteins in black, 12S rRNA in gray.

While the structure of the human mitochondrial ribosome has been elucidated at high resolution, little is known about its early assembly pathway. A recent study determined the kinetics by which ribosomal proteins are incorporated into the mtSSU (Figure S4), predicting that clusters of neighboring proteins were first assembled into modules prior to incorporation into the growing complex.^15^ The specific proteins with reduced expression observed in each of our snMRP^KO^ cell lines are neighboring proteins with close interactions in the mtSSU (Figure 2). Therefore, our data could enable predictions on the composition of the modular units that are assembled into the mtSSU. We used the fold-expression changes of 25 out of the 29 mtSSU proteins that were reproducibly quantified from whole-cell lysates for hierarchical clustering analysis (key resources table) (Figure 3A). We assessed whether the assigned clusters could be inferred as modules by comparing to the positions of ribosomal proteins in the mtSSU structure (Figure 3B). Network analysis revealed four protein clusters, with two clusters associated with the head and platform regions and the other two with the body and foot regions of the mtSSU. Pearson correlation coefficients within these clusters are high, as indicated by the red coloring in the heatmap, suggesting that proteins within each cluster may have a high potential for interaction or functional association. We next assessed whether the expression levels of known mtSSU assembly factors correlate with assembly status of the mtSSU and therefore could allow for potential discovery of novel mtSSU assembly factors. The detected assembly factors were indeed altered in response to specific snMRP knockout (Figure S5). As whole-cell proteomics cannot discern between unbound protein and protein assembled into the mitoribosome, we validated our findings by comparing to mitoribosome assembled fractions. We pooled the fractions corresponding to the mtSSU from sucrose gradients for snMRP^KO^ mS33, mS34, and mS37 for subsequent mass spectrometry analysis. As expected, the sucrose fractions allowed quantification of all mtSSU proteins (with the exception of mS38) showing higher log-fold differences but overall a similar distribution (Figure S6). Whether assembly intermediates lacking individual subunit proteins would be functional in mitochondrial protein synthesis is not known.

### Knockout of mtSSU snMRPs causes distinct translation defects and OXPHOS dysfunction

**Figure 4:**
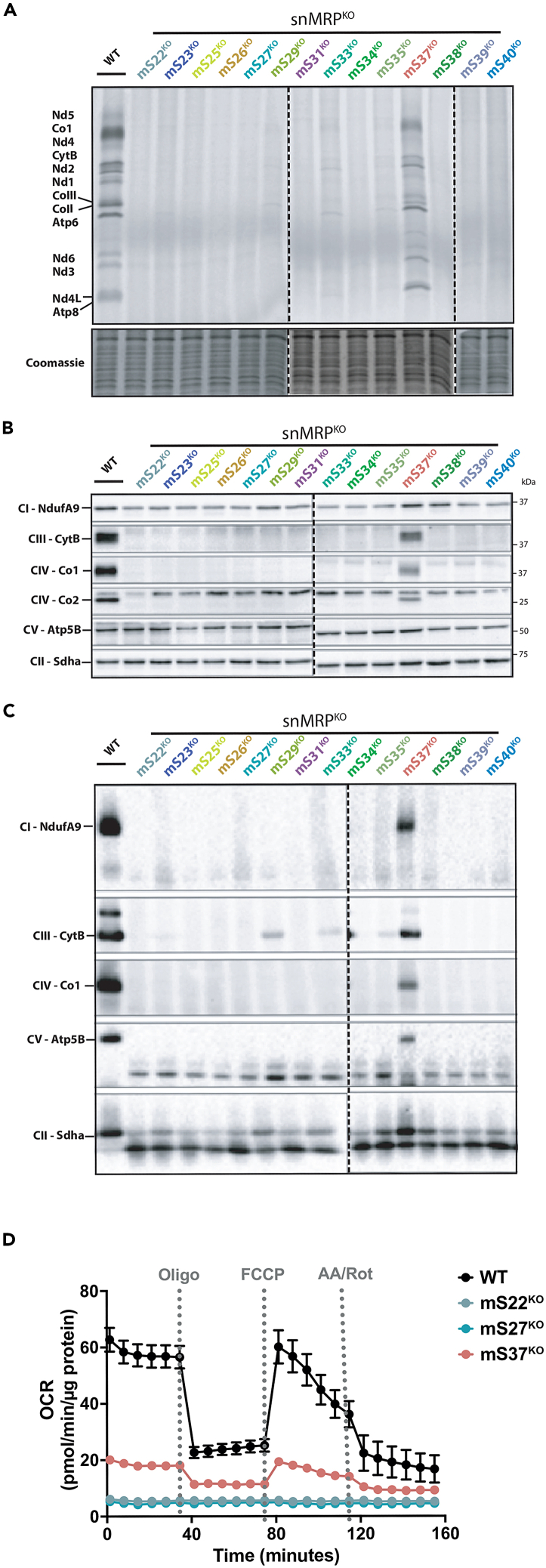
Effect of snMRP knockout on mitochondrial protein synthesis and steady-state levels of OXPHOS complexes and subunits (A) ^35^S-metabolic labeling to assess mitochondrial protein synthesis in snMRP^KO^ lines. (B) Immunoblot of steady-state mitochondrial OXPHOS complex subunits. Asterisks indicate MT-CO2 detection. (C) Immunoblot of native OXPHOS complexes. Dotted line delineates separate gel where controls are excluded for readability. (D) Seahorse high-resolution respirometry in selected snMRP knockout lines. Data are the mean of octuplicate measurements with error bars representing SD.

To address the functional consequences of snMRP deficiency, we used metabolic labeling of ^35^S-methionine/cysteine to analyze mitochondrial protein synthesis. After 60-min pulse labeling, we observed a robust inhibition of mitochondrial protein synthesis for all of the snMRP^KO^ cell lines except mS37^KO^ (Figure 4A). However, an extended labeling reaction for 4 h did reveal low levels of mitochondrial protein synthesis in mS29^KO^, mS33^KO^, and mS35^KO^ cell lines (Figure S7).

Next, we investigated the expression and assembly of OXPHOS complexes, using SDS and blue native PAGE analyses (Figures 4B and 4C) in all our snMRP^KO^ cell lines. These analyses revealed undetectable levels of the assembled complexes in most snMRP^KO^ cell lines with the exception of mS29^KO^, mS33^KO^, and mS35^KO^ cell lines whereby some level of assembled complex III could be detected. mS37^KO^ was the only cell line retaining detectable levels of all assembled complexes and substantial OXPHOS capacity (Figure 4D).

### The stability of mS37 is dependent on oxidizable cysteine residues for regulating mitochondrial protein synthesis

**Figure 5:**
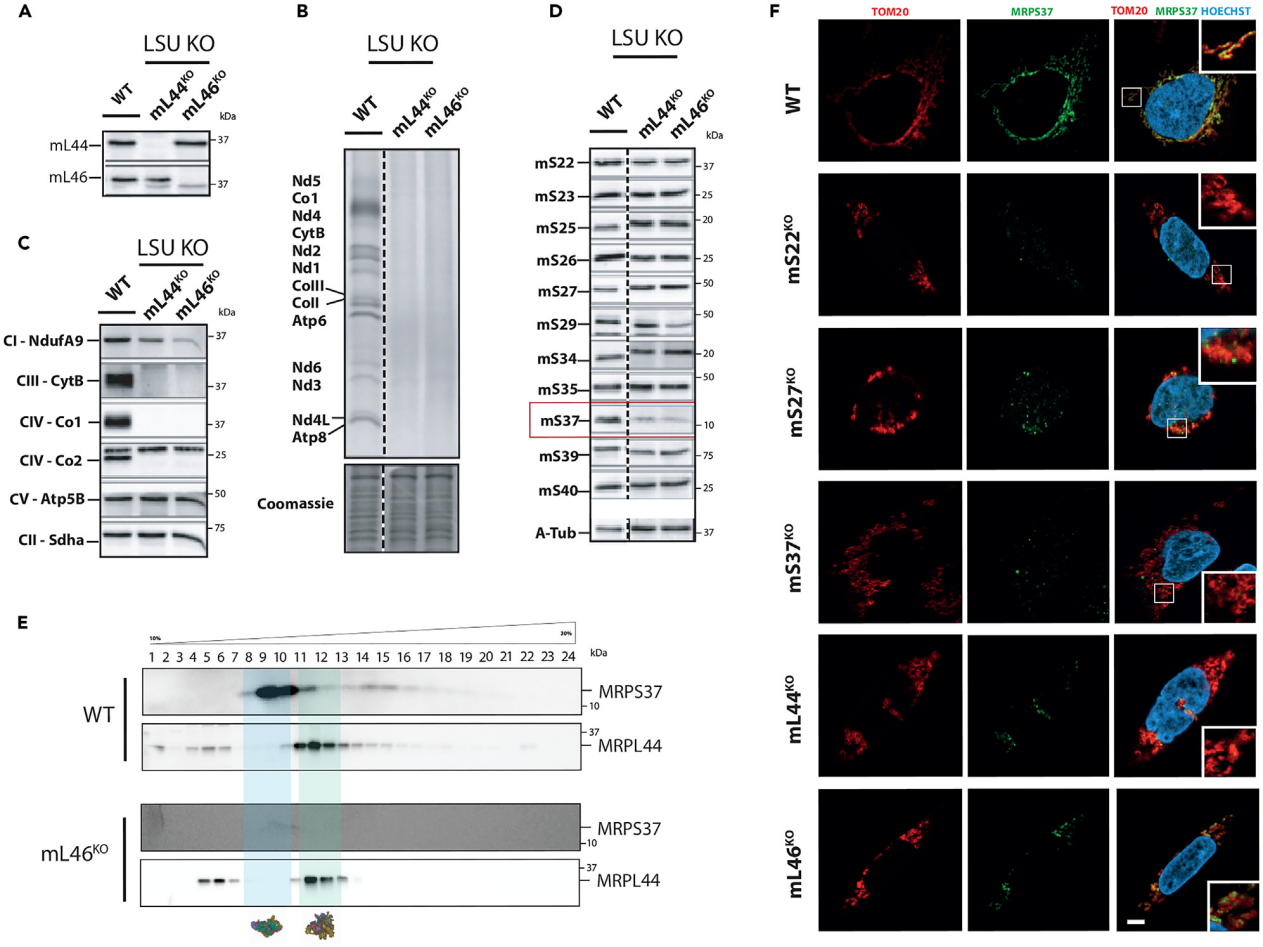
Effect of mtLSU knockouts on mS37 stability (A) Immunoblot confirming CRISPR-mediated mL44^KO^ and mL46^KO^. (B) ^35^S-metabolic labeling to assess mitochondrial protein synthesis in mL44^KO^ and mL46^KO^. (C) Immunoblot for steady state of OXPHOS complex subunits in mL44^KO^ and mL46^KO^. (D) Immunoblot confirming impaired mS37 stability in mL44^KO^ and mL46^KO^. (E) Isokinetic sucrose gradients of mitoribosome assembly in mL46^KO^. (F) Immunofluorescence to assess mitochondrial structure and mS37 localization using antibodies against TOM20 and mS37. DNA stained with Hoechst label. Right panel shows overlay of TOM20, mS37, and Hoechst with inset showing magnified area in box. Scale bar, 2 μm.

Of all the snMRP^KO^ cell lines that we generated, only mS37^KO^ retained substantial mitochondrial protein synthesis and OXPHOS complexes, and yet mS37 stability was reduced in all of these cell lines (Figure 1B). In contrast, the abundance of the other mtSSU proteins was determined by the presence or absence of neighboring interacting proteins. To understand the role of mS37 in mammalian translation further, we generated two knockout cell lines of snMRPs in the mtLSU to test if the protein stability correlated with assembly defects and/or overall defects in mitochondrial protein synthesis (Figure 5A). Knockouts for mL44^KO^, a known disease associated mtLSU protein,^16^ and mL46^KO^ generated a defect in mitochondrial protein synthesis (Figure 5B) and a reduction in the steady-state abundance of mitochondrially encoded OXPHOS complexes (Figure 5C). Immunoblotting against mS37 in these snMRP^KO^ cell lines revealed that, while the mtSSU remains intact in the mtLSU knockouts, the amount of mS37 is reduced (Figure 5D) and is not detectable in fractions representing SSU or LSU intermediates in mL46^KO^ (Figure 5E). A recent report using proximity labeling with BioID suggests that mS37 could be dual localized within the cell.^17^ However, immunofluorescence experiments with mS37 demonstrate only mitochondrial localization (Figure 5F). Together, these findings show that mS37 stability is compromised in assembly defects of both the mtSSU and mtLSU but on its own is not essential for assembly.

**Figure 6:**
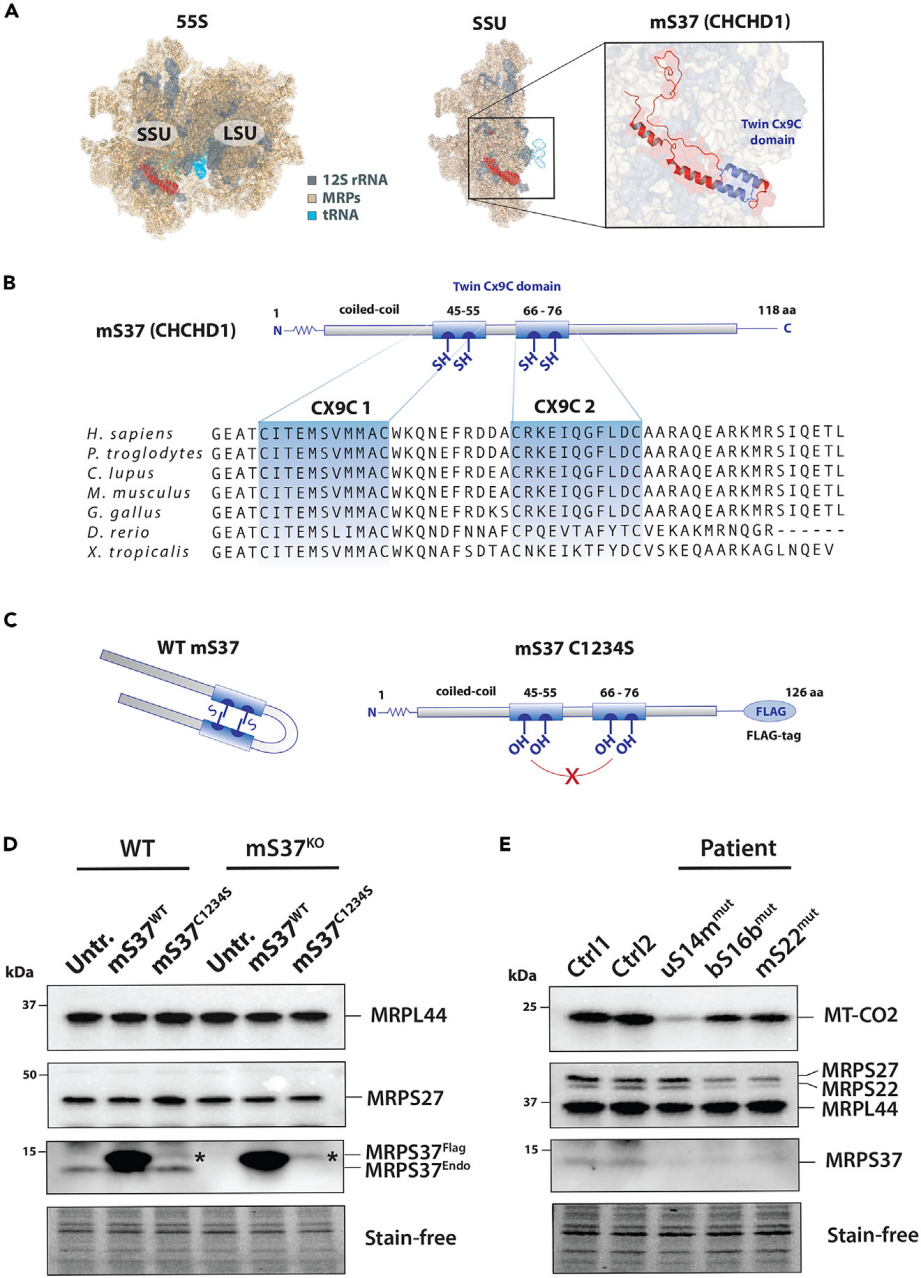
Stability of mS37 is regulated by CX_9_C motif (A) Location of mS37 within the 55S mitoribosome. (B) Conservation of the CHCHD4/MIA40-oxidizable twin CX_9_C motifs in mS37 and (C) subsequent folding. (C) Schematic representation of 4 mutated cysteine residues into serine (C1234S). (D) Immunoblot of FLAG-tagged wild-type and C1234S mS37. Asterisks indicate low levels of C1234S mS37. (E) Immunoblot of mS37 protein level in patient-derived fibroblasts.

One interpretation of the mS37 phenotype is that the protein has a regulatory function in mitochondrial protein synthesis. mS37 has a coiled-coil-helix-coiled-coil-helix domain (CHCHD) containing conserved twin CX_9_C motifs^18^ (Figures 6A and 6B). Mitochondrial import of the CHCHD family members is regulated by the MIA40 receptor, which forms disulfide bonds at the cysteine residues to stabilize the nascent protein during import.^19^ This interaction is essential for mitochondrial protein import with factors that lack classical mitochondrial targeting sequences.^18^ Defects in this redox-mediated regulation lead to protein import failures.^18^^,^^19^^,^^20^ To test whether the CX_9_C motif in mS37 is necessary for protein stability, we mutated all four cysteines to serine within the CX_9_C motif (Figure 6C) and expressed a FLAG-tagged version (mS37 C1234S) in the mS37^KO^ cell line. While wild-type mS37 was stable, the mutant mS37 C1234S was detectable only at very low levels, suggesting the cysteine residues were necessary for protein stability (Figure 6D).

We hypothesized that stability of mS37 would be impaired in diseases associated with variants in mtSSU proteins. Therefore, we examined the stability of mS37 in patient-derived fibroblasts with mitochondrial ribosomal pathogenic variants in the mtSSU (uS14m, bS16b, and mS22).^21^^,^^22^^,^^23^ In all cases, mS37 levels were reduced compared to controls (Figure 6E). Together our results suggest that mS37 is acutely sensitive to defects in mitochondrial protein synthesis and that its stability is regulated via disulfide bonds.

## Discussion

In this study we investigated the importance of the human snMRPs of the mtSSU to mitochondrial ribosome assembly. We show that most of these mitochondria-specific snMRPs are essential for mtSSU assembly and mitochondrial protein synthesis, with the exception of mS37 (MRPS37/CHCHD1). The addition of proteins and concomitant reduction of rRNA in the mammalian mitoribosome can allow for more sophisticated regulation of protein synthesis. Our study highlights the essentiality of supernumerary proteins to mitochondrial protein synthesis. It further supports that—albeit at new positions—snMRPs are essential structural proteins of the mammalian mitoribosome. Moreover, mS37 represents a unique mtSSU protein with a highly specialized role in mitochondrial protein synthesis. Our experiments support that mS37 could act as maturation control of the ribosome prior to the initiation of translation. Recently, cryo-EM structures determined translation pre-initiation steps of the mtSSU and revealed that mS37 and mtIF3 interaction provides a conformation favorable for mtIF2 accommodation,^24^ and mS37 links the final steps of mtSSU assembly and mitochondrial translation initiation.^25^

Besides mS37 there are several proteins with specialized functions for mitochondrial translation such as mL45 that anchors the mitoribosome to the inner membrane and directs the nascent polypeptide toward the tunnel exit securing their delivery to the membrane.^3^ Meanwhile, mS39 serves as a platform for incoming leaderless mRNAs through its pentatricopeptide repeats.^10^ The adaptation of replacing rRNA with protein components in the mitochondrial ribosome likely reflects a strategic evolution to meet the demands of the mitochondrial environment. Mitochondria generate elevated levels of reactive oxygen species as a by-product of OXPHOS. Indeed, it has been raised that the emergence of the snMRPs serves as a protective mechanism, safeguarding the rRNA core from oxidative stress.^10^ These specialized functions underscore the unique adaptation of snMRPs to the mitochondrial environment, highlighting their indispensable role in mitochondrial translation.

Given the fact that mitochondrial translation is a highly energy-consuming process, it would be interesting to consider if there are factors that regulate translation. A recent study showed that the twin CX_9_C motif in mS37 is regulated by the NADH/NAD ratio in the intermembrane space of the mitochondria.^26^ This finding crucially links the mitochondrial translation mechanism to the overall energy status of the cell. Essentially, mS37 acts as a critical checkpoint, controlling the initiation of translation in response to the cell’s energetic needs. Such regulation underscores the intricate balance between cellular energy states and essential biosynthetic activities like protein synthesis in mitochondria. We found oxidizable cysteine residues in a twin CX_9_C motif in mS37 to be critical for its stability as has been shown for NDUFAF8 carrying the same motif.^26^

In our assessment of whole-cell mtSSU protein abundance, we observed that inferred clusters were similar to steady-state protein assembled in the mitoribosome as determined from pooled sucrose fractions, both of which loosely corresponded to the assembly modules previously identified by pulse-labeling proteomic methods.^15^ Interestingly, we found that certain defects in the mtSSU also compromise the integrity of the mtLSU. This observation could align with the known role of mS27 in intersubunit bridging,^27^ offering a plausible explanation for the observed alterations in the mtLSU in our study. This could represent decay and hints to the potential roles of other proteins in preventing premature subunit joining during mitoribosome assembly.

Furthermore, our findings underscore the utility of individual gene knockouts in revealing the intricacies of the mitoribosome assembly pathway, similar to studies performed in yeast.^28^ Our analyses have identified several closely interacting proteins, likely constituting the modular units of the mitoribosome. These results not only corroborate but also refine the predictions made based on the pulse-labeling data from Bogenhagen et al. (2018), enhancing our understanding of mitoribosome assembly.^15^

Although all MRPs seem stably expressed through tissue development, they show differential expression in certain cancers and disease presentations.^23^^,^^29^^,^^30^^,^^31^ This suggests the potential of heterogeneous mitoribosome composition across cell types. Indeed, mutations in mitoribosomal proteins lead to variable disease phenotypes (Table S1).^16^^,^^21^^,^^22^^,^^23^^,^^32^^,^^33^^,^^34^^,^^35^^,^^36^^,^^37^^,^^38^^,^^39^^,^^40^^,^^41^^,^^42^^,^^43^ To date, snMRP proteins associated with human disease are mS22, mS23, mS25, mS34, and mS39.^22^^,^^32^^,^^36^^,^^37^^,^^39^ All reported patients have mitoribosome instability. Although the pathomechanistic consequence is combined OXPHOS deficiency, pathogenic variants in snMRPs lead to distinct phenotypic manifestations. This includes brain abnormalities and hypertrophic cardiomyopathy for mS22,^22^ hepatic disease for mS23,^36^ encephalomyopathy for mS25,^37^ and Leigh syndrome for both mS34^32^ and mS39.^39^

In summary, our study underscores mS37 as a unique protein of the mtSSU in regulating mitochondrial protein synthesis and demonstrates the essential nature of snMRPs for mtSSU assembly and OXPHOS. Furthermore, we identify the composition of proteins that form modular units in the mtSSU assembly pathway.

### Limitations of the study

In our study, we employed knockout models of mitochondrial ribosomal proteins to deduce functional consequences on mitochondrial translation and ribosomal modular assembly. Our methodology incorporated whole-cell label-free proteomics for inferring assembled protein complexes, encountering detectability limitations for certain subunits (e.g., uS10, mS24, mS33, and mS37). A significant limitation of this approach is the reliance on steady-state measurements rather than direct observations of the integral complex assembly, which may not fully capture the dynamic process of mitoribosome assembly. Consequently, our findings primarily reflect the functional necessity of these components in mitochondrial translation without direct insights into the kinetic assembly process mediated by various assembly factors. To address these limitations and advance our understanding of mitochondrial ribosome assembly, future studies should aim to integrate kinetics in combination with structural analysis.

## STAR★Methods

### Key resources table

**Table:** 

REAGENT or RESOURCE	SOURCE	IDENTIFIER
**Antibodies**		

MRPS22	Proteintech	Cat# 10984-1-AP; RRID: AB_2146488
MRPS23	Proteintech	Cat# 18345-1-AP; RRID: AB_2301078
MRPS25	Proteintech	Cat#15277-1-AP; RRID: AB_2180358
MRPS26	Proteintech	Cat# 15989-1-AP; RRID: AB_10858479
MRPS27	Proteintech	Cat# 17280-1-AP; RRID: AB_2180510
DAP3/MRPS29	Proteintech	Cat# 10276-1-AP; RRID: AB_10695891
MRPS34	Proteintech	Cat# 15166-1-AP; RRID: AB_2144233
MRPS35	Proteintech	Cat# 16457-1-AP; RRID: AB_2146521
CHCHD1/MRPS37	Proteintech	Cat# 11728-1-AP; RRID: AB_2079769
PTCD3/MRPS39	Proteintech	Cat# 25158-1-AP; RRID: AB_2879931
MRPS18B/MRPS40	Proteintech	Cat# 16139-1-AP; RRID: AB_2146368
MRPL44	Proteintech	Cat# 16394-1-AP; RRID: AB_2146062
MRPL46	Proteintech	Cat# 16611-1-AP; RRID: AB_2146074
NDUFA9	Abcam	Cat # ab14713; RRID: AB_301431
MT-CYTB	Proteintech	Cat#; 55090-1-AP RRID: AB_2881266
MTCO1	Abcam	Cat# ab14705; RRID:AB_2084810
MT-CO1	Abcam	Cat # ab14607; RRID: AB_2084810
MT-CO2	Abcam	Cat # ab79393; RRID: AB_1603751
ATP5B	Abcam	Cat# ab14748; RRID: AB_301447
SDHA	Abcam	Cat# ab14715; RRID: AB_301433
TOM20	Santa Cruz	Cat# sc-11415; RRID: AB_2207533
Alpha-tubulin	Sigma Aldrich	Cat# T5168; RRID: AB_477579
Alexa488 donkey anti-rabbit	Invitrogen	Cat# A-11001; RRID: AB_2534069
Alexa594 donkey anti-mouse	Invitrogen	Cat# A-21207; RRID: AB_141637
Goat anti-Rabbit, HRP conjugated	Molecular Probes	Cat# G21234; RRID: AB_1500696
Goat Anti-Mouse, HRP conjugated	Jackson ImmunoResearch	Cat# 115-035-146; RRID: AB_2307392

**Chemicals, peptides, and recombinant proteins**		

Oligomycin	Sigma Aldrich	Cat# O4876
FCCP	Sigma Aldrich	Cat# C2920
Antimycin A	Sigma Aldrich	Cat# A8674
Rotenone	Sigma Aldrich	Cat# R8875
DMEM	Sigma Aldrich	Cat# 41965-039
Glutamax	Thermo Fisher Scientific	Cat# 35050038
Uridine	Calbiochem	Cat# 6680
Fetal Bovine Serum (FBS) Heat Inactivated	Sigma Aldrich	Cat# F9665
Fetal Bovine Serum (FBS) dialyzed	Thermo Fisher Scientific	Cat# A3382001
Sodium pyruvate	Thermo Fisher Scientific	Cat# 11360039
EasyTag Express 35S protein labeling mix	PerkinElmer	Cat# NEG772014MC
Anisomycin	Sigma Aldrich	Cat# A9789
Chloramphenicol	Sigma Aldrich	Cat# C3175
Pierce™ Protease Inhibitor Tablets, EDTA-free	Thermo Fisher Scientific	Cat# A32955
Halt™ Phosphatase Inhibitor Cocktail	Thermo Fisher Scientific	Cat# PI78420
SYBR Green Supermix	Bio Rad	Cat# 1725006CUST
Protein Assay Dye Reagent Concentrate	Bio Rad	Cat# 500-0006
VECTASHIELD® Antifade Mounting Medium, With DAPI	Vector Laboratories	Cat# H-1200-10
Dimethyl sulfoxide (DMSO)	Sigma Aldrich	Cat# D8418
ProLong™ Gold Antifade	Thermo Fisher Scientific	Cat# P36934

**Critical commercial assays**		

Maxima First Strand cDNA Synthesis Kit for RT-qPCR	Thermo Fisher Scientific	Cat# K1672
QIAquick PCR purification kit	Thermo Fisher Scientific	Cat# M36008
DirectPCR® DNA Lysis Reagent	VWR	Cat# 732-3255

**Deposited data**		

Mass spectrometry data	PRIDE repository	PXD034224

**Experimental models: Cell lines**		

Human diploid male control fibroblasts	Jackson lab	N/A
MRPS14 (uS14m) patient cell line	Jackson et al., 2019^23^	N/A
MRPS16 (bS16b) patient cell line	Taylor lab	N/A
MRPS22 (mS22) patient cell line	Saada et al., 2007^22^	N/A
HEK293	ATCC	CRL-1573
HEK293 MRPS22^KO^	This study	N/A
HEK293 MRPS23^KO^	This study	N/A
HEK293 MRPS25^KO^	This study	N/A
HEK293 MRPS26^KO^	This study	N/A
HEK293 MRPS27^KO^	This study	N/A
HEK293 MRPS29^KO^	This study	N/A
HEK293 MRPS31^KO^	This study	N/A
HEK293 MRPS33^KO^	This study	N/A
HEK293 MRPS34^KO^	This study	N/A
HEK293 MRPS35^KO^	This study	N/A
HEK293 MRPS37^KO^	This study	N/A
HEK293 MRPS38^KO^	This study	N/A
HEK293 MRPS39^KO^	This study	N/A
HEK293 MRPS40^KO^	This study	N/A
HEK293 MRPL44^KO^	This study	N/A
HEK293 MRPL46^KO^	This study	N/A

**Oligonucleotides**		

mS22 guide 1 cgcccggagaactcctcaag	Sigma	N/A
mS22 guide 2 tcggagctgaaccggcggcg	Sigma	N/A
mS23 guide 1 ccgagagaagatgctcccta	Sigma	N/A
mS23 guide 2 cggtagcagctagtcacgct	Sigma	N/A
mS25 guide 1 agtgaattacaacacgcatg	Sigma	N/A
mS25 guide 2 gttgccgccatgcccatgaa	Sigma	N/A
mS26 guide 1 atggccgagtctcctcactg	Sigma	N/A
mS26 guide 2 gcgagtgaacatgccgcccg	Sigma	N/A
mS27 guide 1 agatggctgcctccatagtg	Sigma	N/A
mS27 guide 2 ggttacagctcgacctctcg	Sigma	N/A
mS28 guide 1 ccgggctccaccttctgtag	Sigma	N/A
mS28 guide 2 tcagtgcctacaccccgaaa	Sigma	N/A
mS29 guide 1 gggaaaagatgtgtgcaaca	Sigma	N/A
mS29 guide 2 acagagtgttcaggtcagtc	Sigma	N/A
mS31 guide 1 atgctactcactgttcggca	Sigma	N/A
mS31 guide 2 tagcccaccggagcgcaaag	Sigma	N/A
mS33 guide 1 gggcactgagacgagacatg	Sigma	N/A
mS33 guide 2 acactgcactgatccatgag	Sigma	N/A
mS34 guide 1 tggactacgagaccttgacg	Sigma	N/A
mS34 guide 2 aggacttgcgcgtgaccagg	Sigma	N/A
mS35 guide 1 caagcggcgttcagtgtcag	Sigma	N/A
mS35 guide 2 gctaggtgtcgggaccggag	Sigma	N/A
mS36 guide 1 cgggactccagtgatcgccg	Sigma	N/A
mS36 guide 2 tttccgcatcttgggcggta	Sigma	N/A
mS37 guide 1 gatgcaagtcgcctctggag	Sigma	N/A
mS37 guide 2 gcttcctcgattgtgccgcg	Sigma	N/A
mS38 guide 1 gcggcggccacaggtcccag	Sigma	N/A
mS38 guide 2 aggaacggccctcaacagct	Sigma	N/A
mS39 guide 1 gcgtgcctcgaccttcagga	Sigma	N/A
mS39 guide 2 ataggactaaggtgactccg	Sigma	N/A
mS40 guide 1 ctgggcgtacgtcaagatgg	Sigma	N/A
mS40 guide 2 cctgaactctgtgagaacct	Sigma	N/A
mL44 guide 1 cattgcacgagagaaaacga	Sigma	N/A
mL44 guide 2 aaccggagggaccagcttgg	Sigma	N/A
mL46 guide 1 cgttctcccacaatgcaccg	Sigma	N/A
mL46 guide 2 aacggcttggagactacagg	Sigma	N/A
U6promFw GAGGGCCTATTTCCCATGATTC	Sigma	N/A
U6promRv GGTGTTTCGTCCTTTCCAC	Sigma	N/A
5pTailedU6promFw GTAAAACGACGGC CAGTGagggcctatttcccatgattc	Sigma	N/A
Term80bpFw gttttagagctaGAAAtagcaag	Sigma	N/A
TermRv80bp AAAAAAAgcaccgactcgg tgccactttttcaagttgataacggactagccttatt ttaacttgctaTTTCtagctctaaaac	Sigma	N/A
3pTailedTerm80bpRv AGGAAACAGCTA TGACCATGAAAAAAAgcaccgactcggtgccac	Sigma	N/A
1_aggc_Fw actgaattcggatcctcGAGCGT CTCACCCTGTAAAACGACGGCCAGT	Sigma	N/A
1_aggc_Rv catgcggccgcgtcgacagatct CGTCTCACATGAGGAAACAGCTA TGACCATG	Sigma	N/A
mS22_seqF aatccctcccaaccacttcc	Sigma	N/A
mS22_seqR cccagcgaaagtccggaa	Sigma	N/A
mS23_seqF gggagaggcagctgcaataat	Sigma	N/A
mS23_seqR ttttggctcggctatcgagt	Sigma	N/A
mS25_seqF gcctcagtctggacctctg	Sigma	N/A
mS25_seqR tacaagtcccagagtgctcc	Sigma	N/A
mS26_seqF ggtcgccgcttcggtt	Sigma	N/A
mS26_seqR caccttcctctgcacctcg	Sigma	N/A
mS27_seqF taggctaaagccgcggatac	Sigma	N/A
mS27_seqR tctaagacccagcaggtggta	Sigma	N/A
mS29_seqF cactagccttttgtgtttcgt	Sigma	N/A
mS29_seqR tccatggtaaattctcaagcaca	Sigma	N/A
mS31_seqF tctaagacccagcaggtggta	Sigma	N/A
mS31_seqR cctttgctgaactctggcga	Sigma	N/A
mS33_seqF acctgggctctattataagaacaa	Sigma	N/A
mS33_seqR gcaaaggaggcaatacagca	Sigma	N/A
mS34_seqF ctgccacagccaggacttg	Sigma	N/A
mS34_seqR tcagcgtcggagtctgagat	Sigma	N/A
mS35_seqF gggggaatcttcctgcacat	Sigma	N/A
mS35_seqR cagacccacgtccatggttt	Sigma	N/A
mS36_seqF acacgtgggtggatcctagt	Sigma	N/A
mS36_seqR tccaacaggctcaaagtccc	Sigma	N/A
mS37_seqF gagcagtcggagtcaggac	Sigma	N/A
mS37_seqR cccaaatgaatgaaagggcct	Sigma	N/A
mS38_seqF ttctgggacctttcggtgcg	Sigma	N/A
mS38_seqR tccctctgtgagcatcgga	Sigma	N/A
mS39_seqF attcctccgaggcaaatcgg	Sigma	N/A
mS39_seqR tgcgttgcgaatcctatttca	Sigma	N/A
mS40_seqF tgcgatctaagagtcgtagtgac	Sigma	N/A
mS40_seqR cgtttagtcctcggctcgg	Sigma	N/A
mL44_seqF cctgccctctctcagtcg	Sigma	N/A
mL44_seqR tgtctccatcgcaaacttcc	Sigma	N/A
mL46_seqF gttctagcagttccgggact	Sigma	N/A
mL46_seqR acggccaatgaaaagaaacca	Sigma	N/A

**Recombinant DNA**		

CAG-Cas9-T2A-EGFP-ires-puro-plasmid	Addgene	#78311
MRPS37^WT^ (OHu15801) pcDNA3.1+/C-(K)-DYK	GenScript	N/A
MRPS37^C1234S^ (OHu15801, p.C45S, p.C55S, p.C66S, p.C76S)	GenScript	N/A

**Software and algorithms**		

Image Lab	Bio Rad	https://www.bio-rad.com/en-fi/product/image-lab-software
Prism 6	GraphPad	https://www.graphpad.com/
CellProfiler	Cimini lab	https://cellprofiler.org/
FlowJo v10	Biosciences	https://www.flowjo.com/solutions/flowjo
Agilent Seahorse Wave	Agilent	https://www.agilent.com

### Resource availability

#### Lead contact

Further information and requests for resources and reagents should be directed to the lead contact (ccarroll@sgul.ac.uk).

#### Materials availability

Several cell lines have been generated in this study, which will be made available upon reasonable request.

#### Data and code availability

##### Data

Data reported in this paper will be shared by the lead contact upon request. Mass-spectrometry proteomics data have been submitted to the ProteomeXchange Consortium via the PRIDE partner repository; Identifier: PXD034224.

##### Code

No original code has been generated in this study.

##### Structural data

No structural data has been generated in this study.

##### Other

Any additional information required to reanalyze the data reported in this paper is available from the lead contact upon request.

### Experimental model and study participant details

Authenticated HEK293 (CRL-1573) cells were obtained from ATCC. Human fibroblasts were obtained from the studies indicated.^22^^,^^23^ Cell lines were routinely tested for mycoplasma infection.

#### Cell culture

Cell lines were grown in DMEM (Gibco) with 4.5 g/l glucose supplemented with 10% heat-inactivated fetal bovine serum, GlutaMax (Sigma, F9665), penicillin/streptomycin, 20 mM pyruvate, 50 uM uridine (Calbiochem, 6680). All cell lines were maintained in a humified chamber at 37°C at 5% CO_2_.

### Method details

#### Design and generation of gRNA-oligos

20 base pair CRISPR-RNA (crRNA) sequences were designed with Benchling (https://www.benchling.com). Two gRNAs per gene were used to preferentially excise the start codon site. The crRNAs were selected according to their off- and on-target score predictions. Testing of the efficiency of crRNAs was performed as described before by the use of a three tailed-template PCR (Balboa et al., 2015). Briefly, the 20 bp crRNAs were combined from the 5’ end to an RNA oligo containing 19 bp matching to a tailed U6 promoter plus an extra G nucleotide (for proper RNA transcription), and from the 3’ end to an RNA oligo containing 19bp matching to a tracRNA and tailed terminator as follows (5’-GTGGAAAGGACGAAACACCgNNNNNNNNNNNNNNNNNNNNGTTTTAGAGCTAGAAATAG -3’). The 59 bp gRNA-oligos were ordered from Sigma. The gRNA-oligos used are listed in the key resources table. Next, U6 promoter containing plasmid pSpCas9(BB)-2A-GFP (PX458) (Addgene, #48138) was digested with the *Bbs*I and *Pvu*I and run on 1% agarose gel. The ∼1.5 kb band was excised from the gel and column-purified. The purified U6 promoter was used as a template in a PCR using Phusion enzyme (ThermoScientific, F530S). The resulting PCR product was column-purified and digested with *Bbs*I overnight to get rid of any possible remaining plasmid. The PCR product was separated on a 1% agarose gel and the 249 bp fragment excised and column-purified.

Using a tailed-PCR-assay, gRNA oligonucleotides were amplified with a U6 promoter and mTERM fragment as previously established.^44^ Tailed U6 was produced by using the digested U6 promoter as a template with primers 5`-tailedU6promFw and U6promRv and tailed terminator was produced by using long reverse oligo TermRv80bp as a template with primers Term80bp and 3`-tailedterm80bpRv in a PCR (primers listed in the key resources table). Resulting products were run on 1 % agarose and the 266 bp PCR product for tailed U6 and 103 bp PCR product for tailed terminator were excised and column-purified. Transcriptional gRNA units were prepared by PCR from gRNA-oligos and tailed U6 and terminators in a PCR.

#### Testing and transfecting cells with gRNAs

Wild-type HEK293 cells were seeded on 24-well plates for ∼ 40% confluency the day before transfections. On the day of transfection, media was changed, and cells were transfected with 500 ng of CAG-Cas9-T2A-EGFP-ires-puro-plasmid (Addgene, #78311) and with 250 ng of gRNAs altogether using PEI transfection reagent with addition of 0.15 M NaCl. The media was changed one day after transfection. Cells were transfected altogether for two days. Transfected cells were FACS-sorted using the co-transfected GFP-CAS9 reporter and knockout clones expanded from GFP-positive FACS-sorted single cells.

#### DNA extraction and PCR confirmation

DNA was extracted from HEK293 cells using DirectPCR® DNA Lysis Reagent (VWR, 732-3255) according to the manufacturer’s instructions. PCR confirmation of the gRNA transfected cells and clonal cell lines were performed in a standard PCR with Sanger sequencing (primers listed in the key resources table).

#### mS37 plasmid transfection

Plasmids expressing wild-type or mutated mS37 cDNAs were purchased from OriGene. HEK293 cells (wild-type or mS37 knock-out) were transfected in 6-well cell culture dish at 70% confluency with 2 μg plasmid using Lipofectamine 2000 (ThermoFisher scientific, Cat#11668027). Cells were collected after 72 hours, lysed with RIPA buffer, and analysed by immunoblotting (see section “immunoblotting”).

#### Immunoblotting

Cells were lysed in 1xPBS containing Pierce™ EDTA-free Protease Inhibitor Mini Tablets (ThermoFisher Scientific, A32955), 1% N-Dodecyl-b-D-Maltoside (Amresco, J424,), 1% Phenylmethanesulfonyl fluoride (PMSF) (Sigma, 93482) on ice for 30 minutes with subsequent centrifugation at 14`000xg at 4°C for 25 minutes. 20 μg of the resulting protein lysates were mixed with 1xLaemmli loading dye containing 5% 2-Mercaptoethanol (Sigma, M3148), separated on 10% polyacrylamide gel and semi-dry blotted to nitrocellulose membrane. The membranes were blocked in TBS-T with 5% milk powder for 1 hour, incubated in primary antibody overnight at 4°C and detected with the respective HRP-secondaries using chemiluminescence. Antibodies are listed in the key resources table.

#### Blue-Native acrylamide electrophoresis

10 cm diameter plates of cells were washed with PBS and scraped into 450 μl of ice-cold 1xPBS. Protein concentrations were determined using Bradford assay and the cells were pelleted by centrifugation in 10`000xg for 10 minutes at 4°C. Cell pellets were resuspended in MB2 buffer, for final protein concentration of 3 μg/μl (for buffers see^45^). n-Dodecyl-β-Maltoside (DDM) was added for a final concentration of 1%, samples incubated on ice for 30 minutes and centrifuged at 20`000xg for 20 minutes at 4°C, after which the supernatant was transferred to a new tube, protein concentration determined, and appropriate loading dye added. 15 μg of protein was separated on NativePAGE 3-12% Bis/Tris gel (ThermoFisher, BN1003BOX) and semi-dry blotted to a nitrocellulose membrane and detected as described above.

#### Translation assays

The cells were grown on 60 mm diameter dishes and labelling was performed at 80 % cell confluency. The cells were washed once with 1xPBS and incubated in pre-warmed labelling medium (DMEM without methionine and cysteine (Sigma, D0422), Glutamax (Gibco, 35050-038), 10% dialysed serum (Thermo Fisher Scientific, A3382001) and 50 μg/ml uridine (Sigma, U3003) for 25 min. 100 μg/ml of anisomycin (Sigma, A9789) was added to each plate 5 minutes prior to addition of labelling mix to inhibit cytoplasmic translation. After that 200 μCi/ml of 35S-methionine/cysteine (PerkinElmer, EasyTag EXPRESS ^35^S Protein Labelling Mix NEG072014MC) was added to each plate and the plates were incubate for 30 minutes. Cells were pulse labelled for 1 or 4 hours after which the cells were washed twice with 1xPBS and scraped into 1 ml of ice-cold 1xPBS. The cells were collected by centrifugation in 14`000xg for 10 minutes at 4°C. The cells were resuspended in ice-cold 1xPBS depending on the pellet size and protein concentration was measured using Bradford. 30 μg of protein was span down by 14`000xg, for 20 minutes at 4°C. The protein pellets were resuspended in 10 μl of H_2_O and 0.3 μl of Benzonase (≥250 units/μl, Sigma, E1014)/sample and the samples were incubated in RT for 5 min. After that 20 μl of 2x loading buffer (186 mM Tris-HCl, pH=6.7-6.8, 15% glycerol, 2% SDS, 0.5 mg/ml bromophenol blue, 6% β-mercaptoethanol) was added and the samples were equilibrated in RT for 1 hour. The samples were run into 12-20% gradient polyacrylamide gel overnight. Next day the gel was rinsed with milliQ-water and dried at 60°C in a gel drier for 75 minutes. The gel was exposed to a phosphor screen and the screen was imaged using Typhoon 9400 scanner.

#### Sucrose gradients

Sedimentation of mitoribosomes was studied by sucrose gradients. Cells were lysed with 1% DDM lysis buffer (50 mM Tris, pH 7.2, 10 mM Mg(CH_3_COO)_2_, 40 mM NH_4_Cl, 100 mM KCl, 1% DDM, 1 mM PMSF, 1.24 mM chloramphenicol, and 1 mM ATP) by incubating the samples for 20 minutes on ice followed by centrifugation at 20`000xg for 20 minutes at 4°C. Protein concentration in the supernatant was determined by Bradford assay and 2 mg of protein of each sample was loaded on top of a 16 ml linear 10-30% sucrose gradient (50 mM Tris, pH 7.2, 10 mM Mg(CH_3_COO)_2_, 40 mM NH_4_Cl, 100 mM KCl, 1 mM PMSF, and 1 mM ATP). The tubes were centrifuged for 15 hours at 4°C and 74`400xg. The gradients were divided into 24 fractions of equal volume from top to bottom, the fractions were TCA precipitated and subsequently used for immunoblotting. For proteomic analysis, the fractions corresponding to the SSU (7-9) were collected and processed as described below.

#### High-resolution respirometry

Mitochondrial respiration was measured using an XFe96 Extracellular Flux Analyzer (Agilent). Cells were cultured in assay-specific 96-well culture plates optimized for cell density (8`000 cells/well). One hour prior to measurement, culture medium was replaced with XF DMEM assay medium (Agilent, 103680-100), supplemented with 10 mM glucose (Sigma, G8270,), 1 mM sodium pyruvate (ThermoFisher Scientific, 11360070,), and 2 mM L-glutamine (ThermoFisher Scientific, 25030123) after which cells were incubated in a non-CO_2_ 37°C incubator. The assay consisted of assessment of basal respiration, after which 1.5 μM oligomycin (Sigma, O4876) was added to measure leak respiration, 1.125 μM carbonylcyanide-4-(trifluoromethoxy)-phenylhydrazone (FCCP (Sigma, C2920)) to measure maximal uncoupled respiration) and a mixture of 1.0 μM antimycin A (Sigma, A8674) and rotenone (Sigma, R8875) to measure residual respiration.

#### Immunofluorescence experiments and analysis

Cells were cultured on coverslips and fixed with 4% paraformaldehyde for 10 minutes. The fixed samples were then washed with 1xPBS for 10 minutes. The cells were permeabilised with 0.1% TritonX-100 for 30 minutes, blocked in 10% horse serum and washed with PBS for 5 minutes. The samples were incubated overnight at 4°C with the primary antibodies: mouse anti-TOM20 (SantaCruz, sc-11415, 1:400), rabbit anti-MRPS37/mS37 (Proteintech, 11728-1-AP, 1: 400). Next, the cells were washed with PBS for 5 minutes and incubated for 30 minutes with the following secondary antibodies: goat anti-rabbit AlexaFluor488 (Invitrogen Antibodies, A-21207, 1: 500) and goat anti-mouse AlexaFluor594 (Invitrogen Antibodies, A-21206, 1: 500). The cells were washed twice with 1xPBS for 5 minutes, stained with 1xPBS containing Hoechst for 5 minutes, washed twice with 1xPBS for 15 minutes and observed with the Zeiss Axio Observer Z1 inverted phase contrast fluorescence microscope with the peak emission wavelengths of 618 nm (red), 517 nm (green) and 465 nm (blue). Mitochondrial fragmentation and co-localization analysis were performed with a minimum of 100 cells per group.

#### Label-free quantitative proteomics

Proteomic analysis was performed in quadruplicates for each cell line. Briefly, each sample was lysed with RIPA-buffer, proteins were precipitated with acetone and trypsin-digested using sequencing grade-modified trypsin (Promega). The resulting peptide mixture was purified by STAGE-TIP method using a C18 resin disk (3M Empore) before the samples were analysed by nanoLC-MS/MS using QExactive HF (Thermo) coupled to nEASY-LC (Thermo). MS raw files were submitted to MaxQuant software v.1.6.1.0 for protein identification and label-free quantification. Carbamidomethyl (C) was set as a fixed modification and acetyl (protein N-term), carbamyl (N-term) and oxidation (M) were set as variable modifications. First search peptide tolerance of 20 ppm and main search error 4.5 ppm were used. Trypsin without proline restriction enzyme option was used, with two allowed miscleavages. The minimal unique + razor peptides number was set to 1, and the allowed FDR was 0.01 (1%) for peptide and protein identification. Label-free quantitation was employed with default settings. UniProt database with ‘Human’ entries (2018) was used for the database searches. Known contaminants as provided by MaxQuant and identified in samples were excluded from further analysis. Perseus software v.1.6.1.3 was used for statistical analysis of the label-free quantification data. The mass spectrometry proteomics data have been deposited to the ProteomeXchange Consortium via the PRIDE partner repository with the dataset identifier PXD034224.

### Quantification and statistical analysis

#### Statistics and software

All graphical representations were performed in Adobe Illustrator and Prism v10.0.1. Structural representations were created using Pymol v.2.4.1. The hierarchical cluster matrix was generated using Python 3.11, with data visualization implemented via Matplotlib 3.8. Pearson correlation coefficients were calculated to assess linear relationships between variables, and these coefficients were used as the basis for clustering. Euclidean distances between Pearson correlations served as the metric for hierarchical clustering. The assigned modules within the hierarchical clustering were defined using the Louvain method.

## Supplemental information

Document S1. Figures S1–S7 and Table S1

Data S1. Whole-cell proteomic profiling of HEK293 WT, MRPS22, and MRPL44, related to Figures 2 and 3

Data S2. Whole-cell proteomic profiling of HEK293 WT, MRPS23-40, and MRPL46, related to Figures 2 and 3

Data S3. SSU pooled sucrose fraction proteomic profiling of HEK293 WT, mS33, mS34, and mS37, related to Figure 3
