# Supplementary material for: Supernumerary proteins of the human mitochondrial ribosomal small subunit are integral for assembly and translation
Source: iScience. 2024 Jun 4;27(7):110185. doi: 10.1016/j.isci.2024.110185 (PMC11251090; doi:10.1016/j.isci.2024.110185)
Supplement: Document S1. Figures S1–S7 and Table S1 [file mmc1.pdf]

iScience, Volume *27*

Supplemental information

Supernumerary proteins of the human mitochondrial ribosomal small subunit are integral for assembly and translation

Taru Hilander, Ryan Awadhpersad, Geoffray Monteuuis, Krystyna L. Broda, Max Pohjanpelto, Elizabeth Pyman, Sachin Kumar Singh, Tuula A. Nyman, Isabelle Crevel, Robert W. Taylor, Ann Saada, Diego Balboa, Brendan J. Battersby, Christopher B. Jackson, and Christopher J. Carroll

Supplementary Figures and Tables

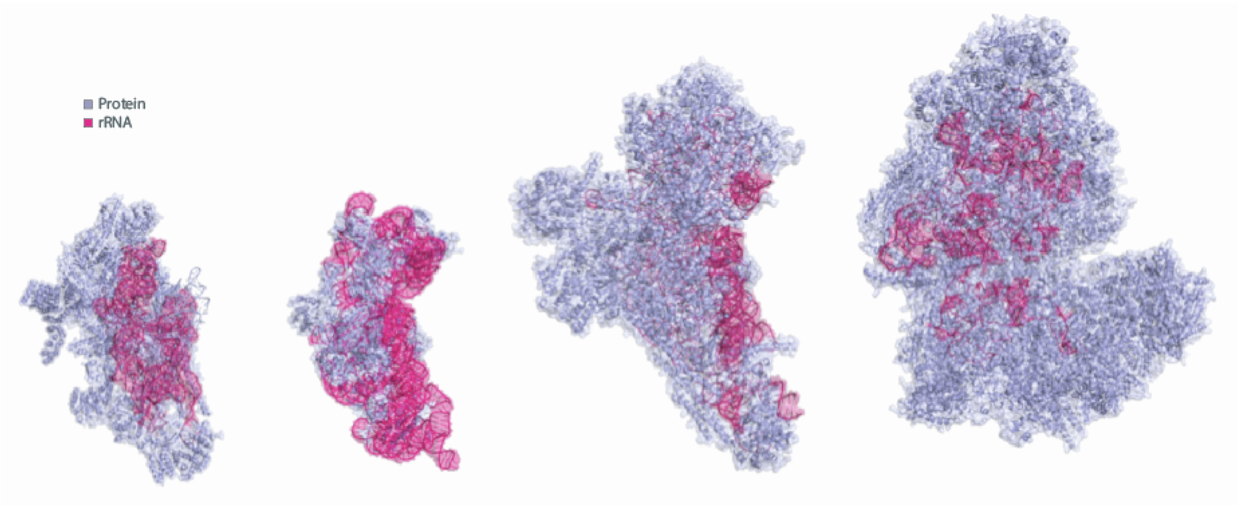

Supplementary Figure S1 (related to Figure 1) ***Comparison of protein-to-RNA composition in the SSU of (mitochondrial) ribosomes.***

From left to right: 28S mammalian mitoribosome, 30S bacterial ribosome, 37S yeast mitoribosome, 78S fungi *Neurospora crassa* (full ribosome).

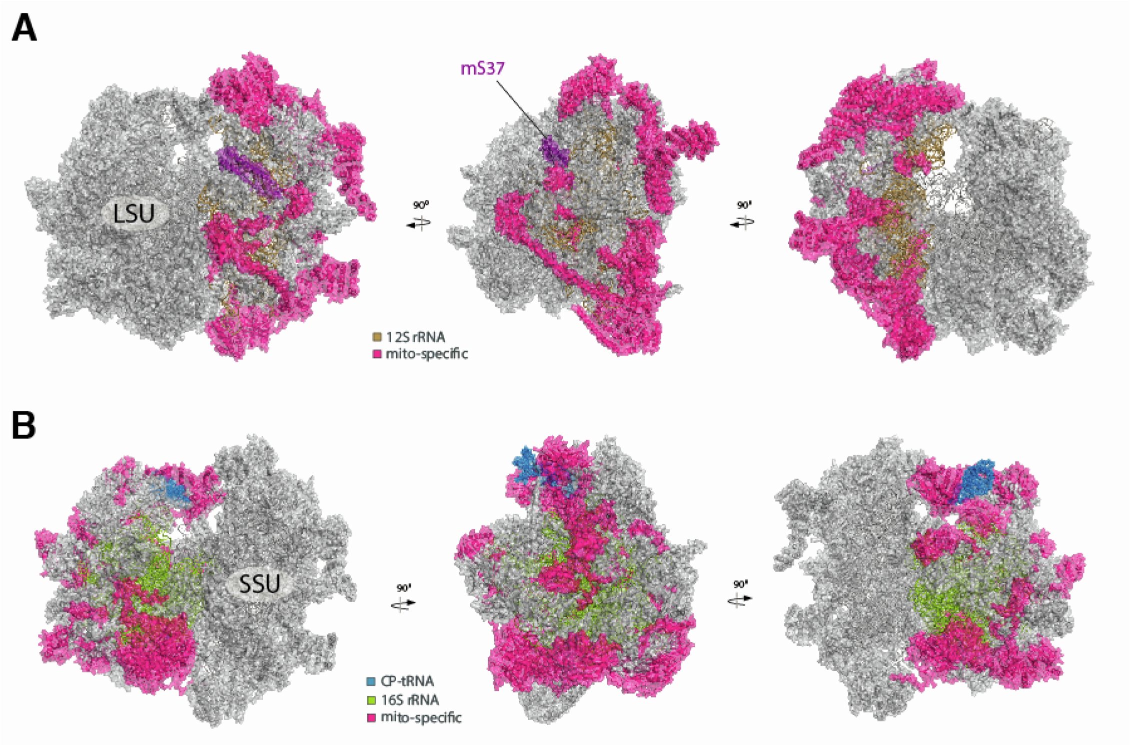

Supplementary Figure S2 (related to Figure 1) ***Mammalian mtSSU (A) and mtLSU (B).***

(**A**) Location of snMRPs within the mtSSU.
(**B**) Location of snMRPs within the mtLSU.

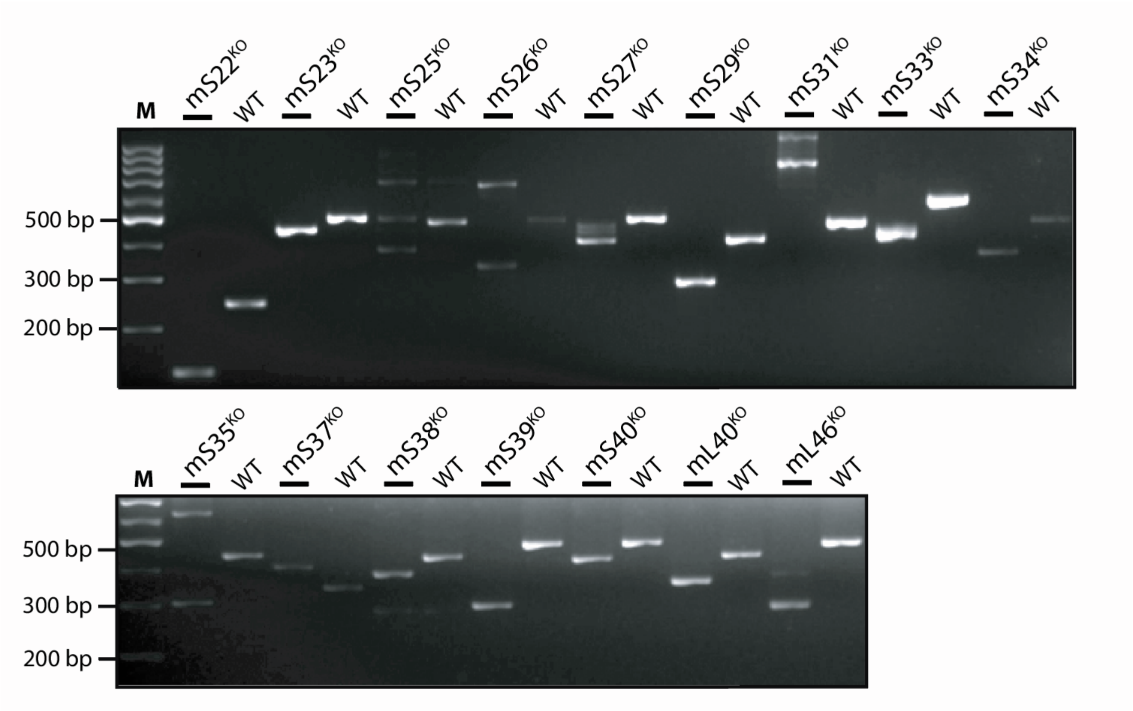

Figure S3 (related to Figure 1) ***Genomic DNA amplification of CRISPR-edited genetic loci.*** The respective primer sequences are listed in the Key resources table.

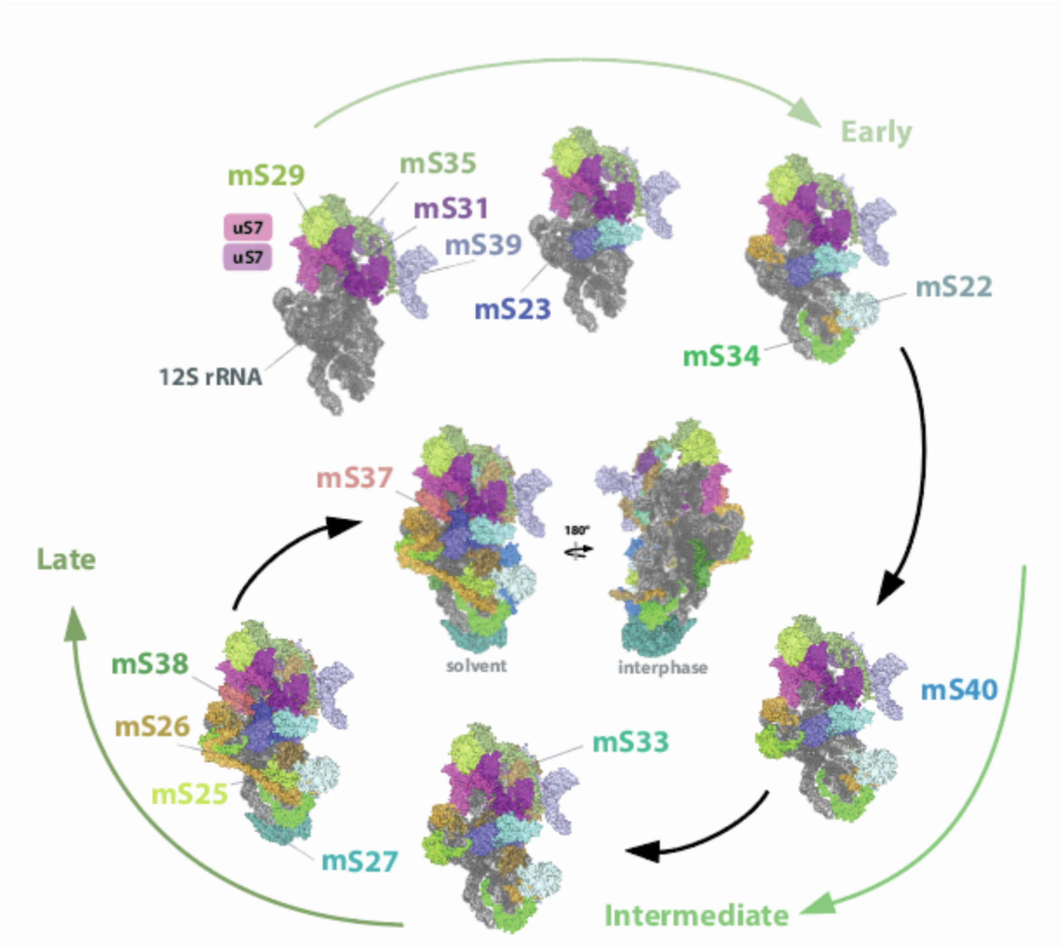

Figure S4 (related to Figure 2) ***Representation of mtSSU assembly pathway with snMRPs highlighted*** (adapted from [6, 9, 15]).

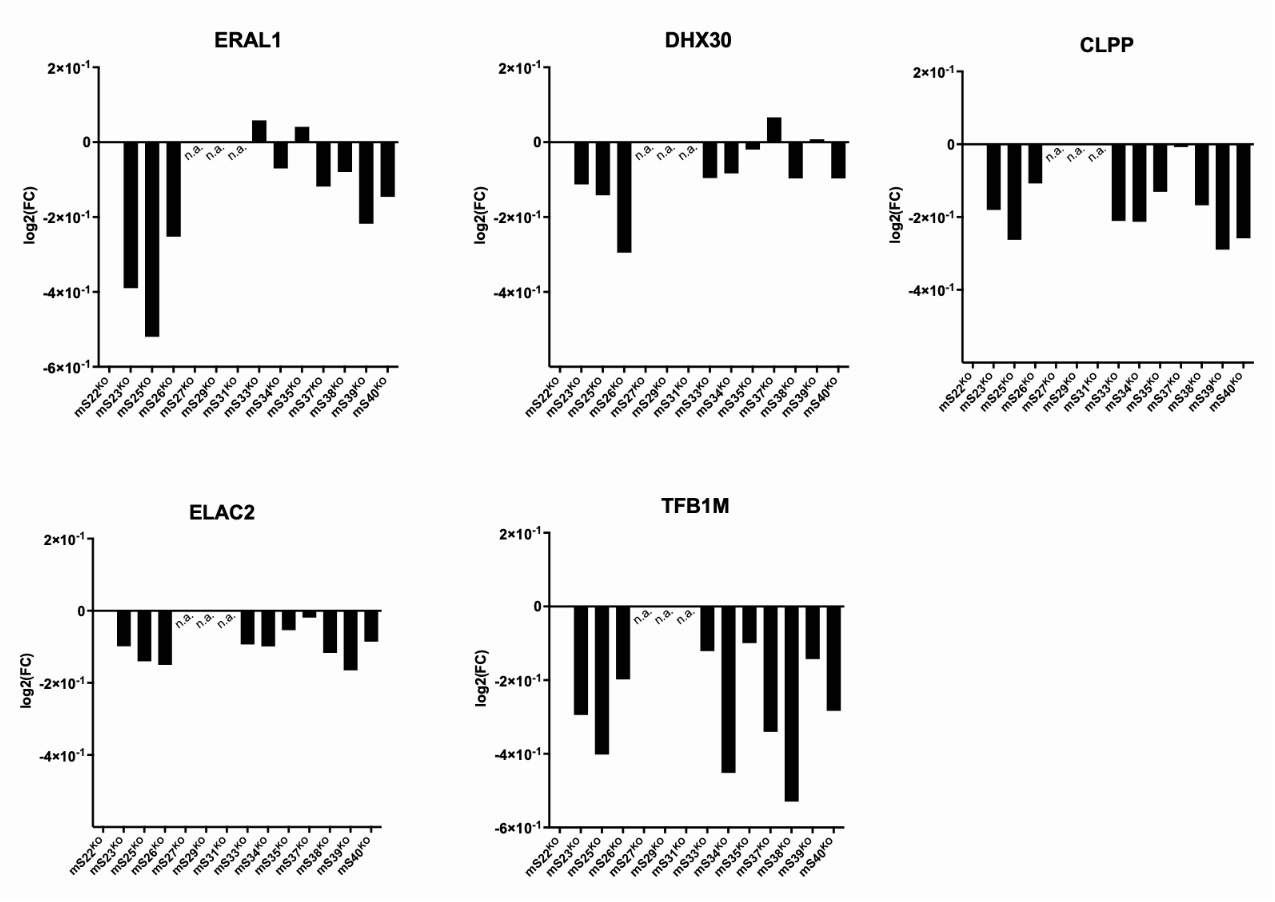

Figure S5 (related to Figure 3) Ribosome assembly and modifying factors levels from proteomic data. Values represent mean of quadruplicate measurements normalised to control.

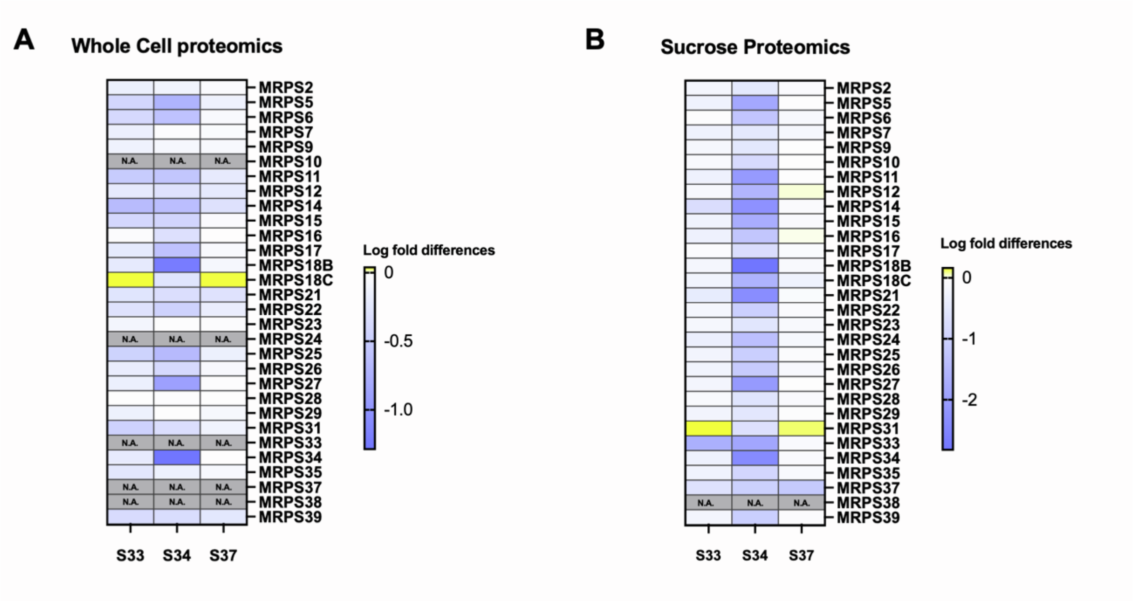

Figure S6 (related to Figure 3) Comparison of label-free quantitative proteomic data from all SSU MRPs from (A) whole cell and (B) pooled sucrose fractions corresponding to assembled mtSSU. (**A**) Quantitative label-free proteomics data of mtSSU protein expression from whole-cell, snMRP knockouts compared to wild-type. Heatmap represents log fold differences and the mean of quadruplicates. The decrease/increase in expression is visualized by a gradient of blue/yellow (scaling shown). Data not available is greyed out. (**B**) Quantitative label-free proteomics data of mtSSU protein expression from pooled sucrose fractions corresponding to the mtSSU, snMRP knockouts compared to wild-type. The decrease/increase in expression is visualized by a gradient of blue/yellow (scaling shown). Heatmap represents log fold differences and the mean of quadruplicates.

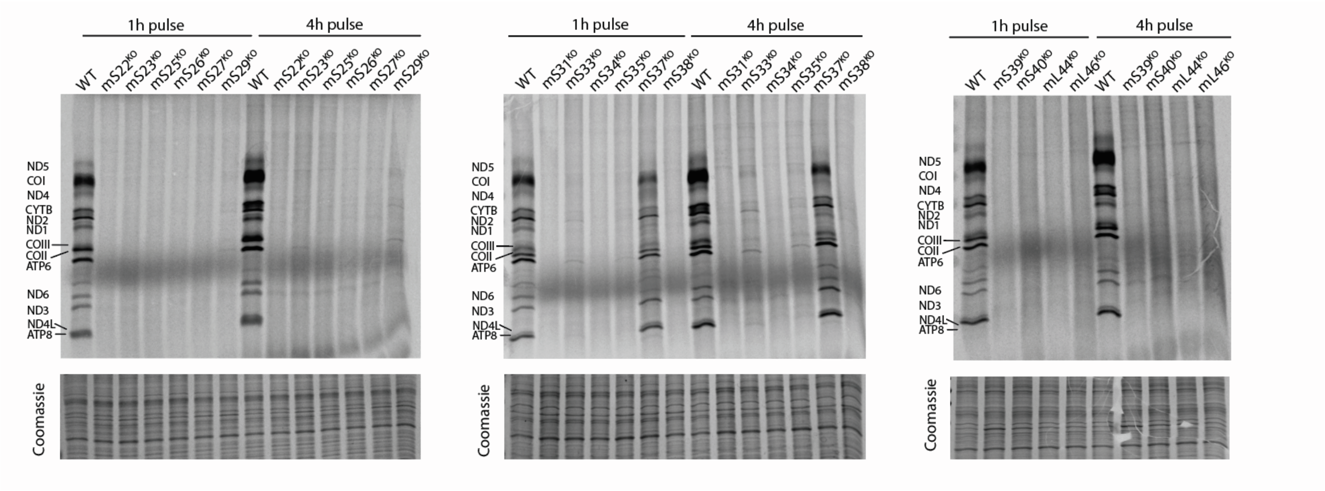

Figure S7 (related to Figure 4) Extended ^{35}S-metabolic pulse labelling.

Table S1. Mitochondrial disease-associated mutations in mitoribosomal proteins.

mtSSU

Subunit / Gene	References
uS2m / MRPS2	[34]
uS7m / MRPS7	[35]
uS14m / MRPS14	[23]
uS16m / MRPS16	[21]
**mS22 / MRPS22***	[22]
**mS23 / MRPS23***	[36]
**mS25 / MRPS25***	[37]
bS1m / MRPS28	[33]
**mS34 / MRPS34***	[32]
**mS39 / MRPS39***	[39]

*** supernumerary subunits**

mtLSU

Subunit / Gene	References
uL3m / MRPL3	[40]
bL12m / MRPL12	[41]
uL24m / MRPL24	[42]
**mL39 / MRPL39***	[38]
**mL44 / MRPL44***	[16]
**mL50 / MRPL50***	[43]

*** supernumerary subunits**
